# The Tumor Microenvironment Mediates the HIF-1α/PD-L1 Pathway to Promote Immune Escape in Colorectal Cancer

**DOI:** 10.3390/ijms25073735

**Published:** 2024-03-27

**Authors:** Jing Sun, Zhengtian Zhao, Jiaqi Lu, Wen An, Yiming Zhang, Wei Li, Li Yang

**Affiliations:** 1 State Key Laboratory of Fine Chemicals, Department of Pharmaceutical Engineering, School of Chemical Engineering, Dalian University of Technology, No. 2, Linggong Road, Ganjingzi District, Dalian 116024, China; sj19980916@mail.dlut.edu.cn (J.S.); 894204239@mail.dlut.edu.cn (Z.Z.); lujiaqi517@mail.dlut.edu.cn (J.L.); lwjieshou@163.com (W.A.); yimingjingren1998@163.com (Y.Z.); liweilw6@126.com (W.L.); 2 Ningbo Institute of Dalian University of Technology, No. 26, Yucai Road, Jiangbei District, Ningbo 315016, China

**Keywords:** colorectal cancer, hypoxic environment, inflammatory environment, HIF-1α, PD-L1, curcumin

## Abstract

The unsatisfactory efficacy of immunotherapy for colorectal cancer (CRC) remains a major challenge for clinicians and patients. The tumor microenvironment may promote CRC progression by upregulating the expression of hypoxia-inducing factor (HIF) and PD-L1. Therefore, this study explored the expression and correlation of HIF-1α and PD-L1 in the CRC microenvironment. The expression and correlation of HIF-1α and PD-L1 in CRC were analyzed using bioinformatics and Western blotting (WB). The hypoxia and inflammation of the CRC microenvironment were established in the CT26 cell line. CT26 cells were stimulated with two hypoxia mimics, CoCl_2_ and DFO, which were used to induce the hypoxic environment. Western blotting was used to assess the expression and correlation of HIF-1α and PD-L1 in the hypoxic environment.LPS stimulated CT26 cells to induce the inflammatory environment. WB and bioinformatics were used to assess the expression and correlation of TLR4, HIF-1α, and PD-L1 in the inflammatory environment. Furthermore, the impact of curcumin on the inflammatory environment established by LPS-stimulated CT26 cells was demonstrated through MTT, Transwell, molecular docking, network pharmacology and Western blotting assays. In this study, we found that the HIF-1α/PD-L1 pathway was activated in the hypoxic and inflammatory environment and promoted immune escape in CRC. Meanwhile, curcumin suppressed tumor immune escape by inhibiting the TLR4/HIF-1α/PD-L1 pathway in the inflammatory environment of CRC. These results suggest that combination therapy based on the HIF-1α/PD-L1 pathway can be a promising therapeutic option and that curcumin can be used as a potent immunomodulatory agent in clinical practice.

## 1. Introduction

Colorectal cancer (CRC) stands out as a common gastrointestinal malignancy globally [1]. In 2020, the number of new cancer cases worldwide exceeded 19 million, with CRC representing 10% of these cases [2]. At the same time, both the incidence and fatality rates of CRC are continuously rising each year. As the majority of patients are typically diagnosed during the mid- to late stages, the therapeutic effects of surgical resection combined with radiotherapy and chemotherapy are unsatisfactory, and the prognosis remains poor [3]. In addition to these conventional therapeutic approaches, the clinical management of CRC has extended to checkpoint blockade of PD-1/PD-L1, i.e., programmed death ligand 1. However, since the efficacy of immune checkpoints can be affected by the complicated tumor microenvironment [4], many patients present with different degrees of immune tolerance after receiving immunotherapy drugs, which reduces the efficacy of immunotherapy [5]. Gaining a more profound understanding of the mechanisms driving immune evasion induced by PD-L1 in CRC holds the potential to enhance the efficacy of immunotherapy interventions.

PD-L1 is a type I transmembrane protein with an extracellular N-terminal domain that promotes tumor cell immune escape and inhibits T cell activation by binding to the T cell PD-1 receptor [6]. Clinical data indicated that many human solid tumors express PD-L1 and that a high level of PD-L1 predicts an unpromising prognosis [7]. Recent studies have shown that Hypoxia-inducible factor 1α (HIF-1α) can stimulate PD-L1 expression in malignant solid tumors, like non-small cell lung cancer [8], hepatocellular carcinoma [9], and glioma [10], which mediates tumor immune escape. However, the correlation between HIF-1α and PD-L1 in CRC has not been reported.

HIF-1α, a key transcription factor in the hypoxic environment of CRC, regulates CRC cell migration [11]. The HIF-1α subunit undergoes rapid degradation in normal oxygen conditions [12]. However, with decreasing oxygen levels, there is a marked elevation in HIF-1α expression, leading it to translocate into the nucleus, where it associates with the HIF-1β subunit to create a dimeric complex [13]. The complex can associate with hypoxia response elements (HREs), impacting CRC development and inducing tumor immune escape. Interestingly, in addition to the hypoxic environment, inflammation can activate HIF-1α in normal oxygen conditions [14]. Lipopolysaccharide (LPS) stands as a robust pro-inflammatory agent. Existing as a constituent of the cell wall in Gram-negative bacteria, it is widely distributed throughout the colon. Both regular and cancerous intestinal cells are continually subjected to its influence [15]. In addition, in vitro assays have shown that LPS can induce CRC development and tumor immune escape [16,17]. Therefore, we hypothesized that the CRC microenvironment may regulate immune escape in CRC by modulating HIF-1a activation.

In this study, we investigated the potential correlation between HIF-1α expression and PD-L1 expression in CRC. We found that a hypoxic and inflammatory environment can activate the HIF-1α/PD-L1 pathway. Meanwhile, we found that TLR4 can regulate the HIF-1α/PD-L1 pathway in the inflammatory environment. Furthermore, we also identified curcumin, a “medicine food homology” anti-inflammatory drug, which can inhibit the development of CRC via the TLR4/HIF-1α/PD-L1 pathway.

## 2. Results

### 2.1. Expression Levels of HIF-1α and PD-L1 in CRC Changed in Parallel

**Figure 1:**
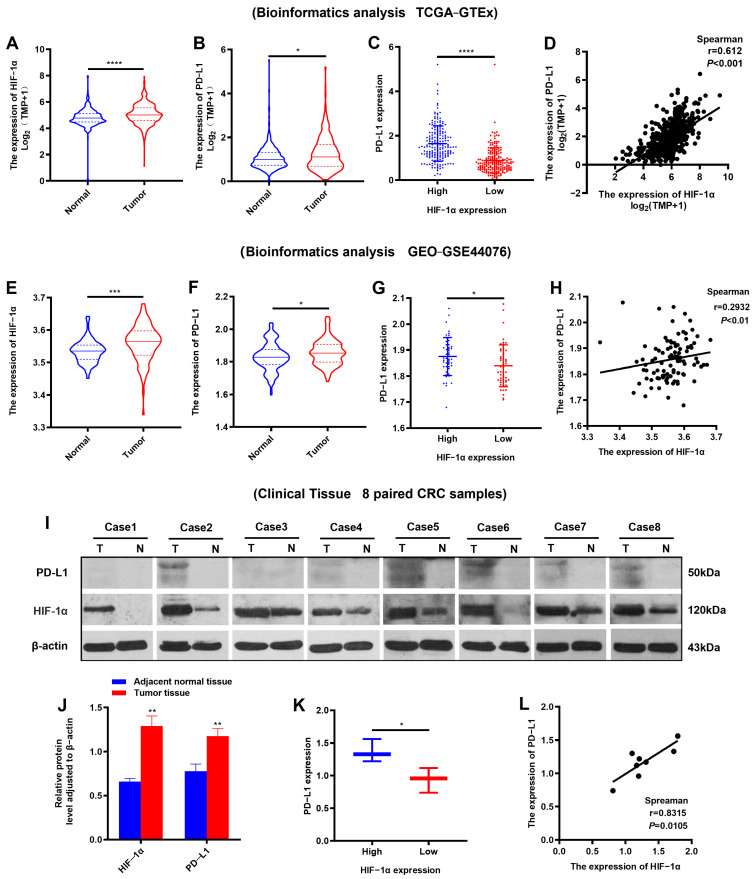
The expression and correlation of HIF-1α and PD-L1 in CRC. (**A**,**B**) The HIF-1α and PD-L1 expression levels of tumor samples and normal samples in CRC cohorts from the TCGA and GTEx databases. (**C**,**D**) The correlation of HIF-1α and PD-L1 in tumor samples in the CRC cohort from the TCGA database (r = 0.612). (**E**,**F**) The HIF-1α and PD-L1 expression levels of tumor samples and normal samples in the gene chip GSE44076. (**G**,**H**) The correlation of HIF-1α and PD-L1 in tumor samples in the gene chip GSE44076 (r = 0.2932). (**I**,**J**) The expression of HIF-1α and PD-L1 determined by Western blotting in CRC samples (*n* = 8). T, tumor tissue; N, adjacent normal tissue. (**K**,**L**) The correlation of HIF-1α and PD-L1 in tumor tissue from CRC samples (r = 0.8315). * *p* < 0.05, ** *p* < 0.01, *** *p* < 0.001, **** *p* < 0.0001.

We performed bioinformatics and clinical paired CRC samples to evaluate the expression levels of HIF-1α and PD-L1, as well as the relevance of HIF-1α and PD-L1 to CRC. Firstly, through analyzing RNA-seq data in the TCGA and GTEx datasets, notable increases in the expression of HIF-1α and PD-L1 were observed in tumor samples compared to normal samples, and the expression of HIF-1α was positively correlated with PD-L1 in tumor samples (Figure 1A–D). In addition, these findings were supported by the validation cohort GSE44076 from the GEO dataset (Figure 1E–H). Further, PD-L1 and HIF-1α levels in eight paired CRC samples were detected using Western blotting (WB). The WB results demonstrated that the expression levels of HIF-1α and PD-L1 were significantly elevated in tumor tissues compared to adjacent normal tissues, and the high expression of HIF-1α was often accompanied by the high expression of PD-L1 in tumor tissues (Figure 1I–L). In summary, these results suggested that HIF-1α and PD-L1 were overexpressed in CRC, and HIF-1α was positively correlated with PD-L1 in CRC.

### 2.2. HIF-1α Upregulated PD-L1 Protein Level in the Hypoxic Environment of CRC

**Figure 2:**
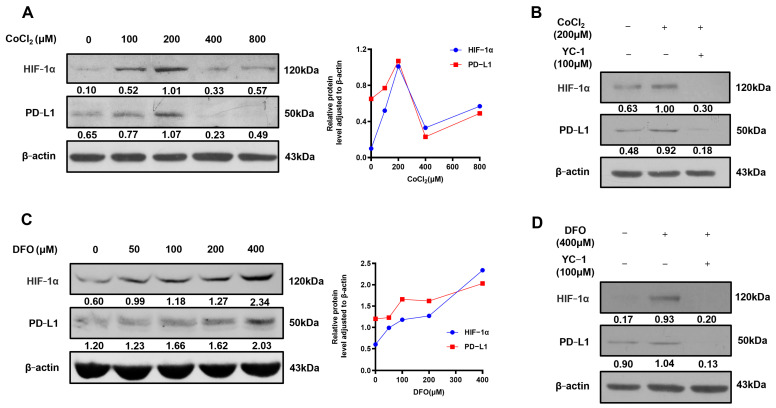
HIF-1α upregulated PD-L1 expression in CT26 cells. (**A**) HIF-1α and PD-L1 protein levels in CT26 cells exposed to various concentrations of CoCl_2_ (0, 100, 200, 400, and 800 μM) for 48 h. (**B**) The expression of HIF-1α and PD-L1 in CT26 cells in the presence or absence of CoCl_2_ (200 μM) after HIF-1α inhibitor YC-1 (100 μM) treatment for 48 h. (**C**) HIF-1α and PD-L1 protein levels in CT26 cells exposed to various concentrations of DFO (0, 50, 100, 200, and 400 μM) for 24 h. (**D**) The expression of HIF-1α and PD-L1 in CT26 cells in the presence or absence of DFO (400 μM) after HIF-1α inhibitor YC-1 (100 μM) treatment for 24 h. One experiment out of three independent experiments is shown.

The hypoxic environment is an important feature of CRC [18]. HIF-1α plays a pivotal role in the hypoxic environment and affects immune escape in certain solid tumors [19], but the response of PD-L1 to HIF-1α in hypoxia remains uncertain in CRC. Therefore, the hypoxic environment was established by stimulating CT26 cells with two hypoxia mimics, CoCl_2_ and DFO, to uncover the related mechanisms. The results of WB revealed that stimulation of CT26 cells with CoCl_2_ or DFO led to a concentration-dependent increase in HIF-1α and PD-L1 levels, showing a maximum upregulation of HIF-1α and PD-L1 at 200 μM CoCl_2_ or 400 μM DFO (Figure 2A,C). To explore whether the PD-L1 expression induced by CoCl_2_ or DFO in CT26 cells depends on HIF-1α, the HIF-1α inhibitor YC-1 was used. The results of WB showed that the YC-1 treatment significantly decreased PD-L1 expression in CT26 cells under hypoxic conditions stimulated by 200μM CoCl_2_ or 400μM DFO (Figure 2B,D). Overall, these data indicated that HIF-1α upregulated PD-L1 in the hypoxic environment of CRC.

### 2.3. TLR4 Regulated HIF-1α/PD-L1 Expression in the Inflammatory Environment of CRC

**Figure 3:**
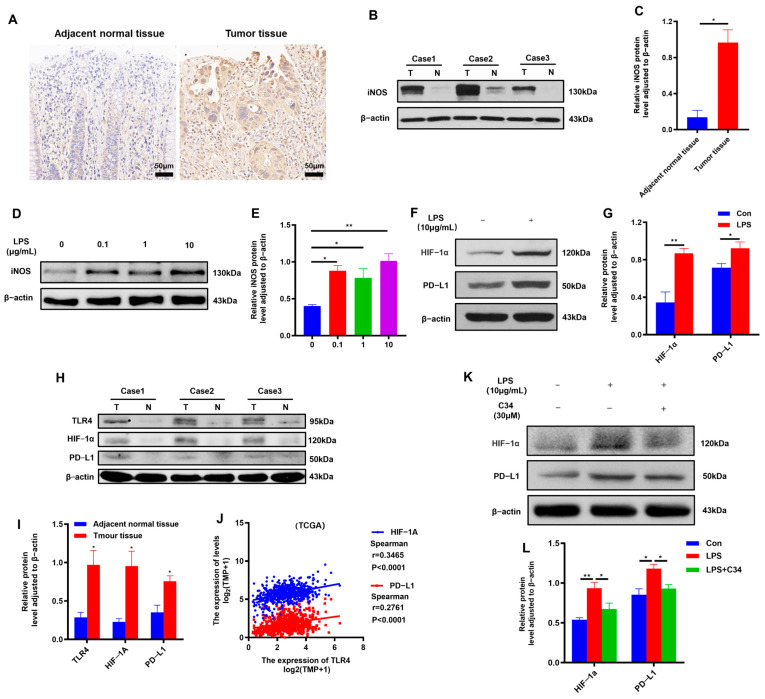
TLR4 regulated HIF-1α/PD-L1 expression. (**A**) Representative images of TLR4 immunohistochemical (IHC) staining in clinical CRC samples (scale bars = 50 μm). (**B**,**C**) The expression iNOS determined by Western blotting in CRC samples (*n* = 3). T, tumor tissue; N, adjacent normal tissue. (**D**,**E**) The iNOS protein level in CT26 cells treated with varying concentrations of LPS (0, 0.1, 1, and 10 μg/mL) for 24 h (*n* = 3). (**F**,**G**) The expression of HIF-1α and PD-L1 in CT26 cells with or without LPS (10 μg/mL) exposure (*n* = 4). (**H**,**I**) The expression of TLR4, HIF-1α, and PD-L1 determined by Western blotting in CRC samples (*n* = 3). T, tumor tissue; N, adjacent normal tissue. (**J**) The correlation of TLR4 with HIF-1α/PD-L1 in tumor samples in the CRC cohort from the TCGA database. (**K**,**L**) The expression of HIF-1α/PD-L1 in CT26 cells with or without exposure to LPS after TLR4 inhibitor C34 (30 μM) treatment for 24 h (*n* = 3). * *p* < 0.05, ** *p* < 0.01.

In addition to the hypoxic environment, the inflammatory environment is also an important feature of CRC [20]. LPS, as an important pro-inflammatory factor in CRC, can activate the TLR4 pathway to regulate the expression of inflammatory cytokines, such as COX2 and iNOS [21]. This is consistent with our clinical results (Figure 3A–C). In addition, the LPS-induced inflammatory environment promoted the expression of HIF-1a, thereby affecting metastasis in cancer [22]. However, whether the LPS-induced inflammatory environment can activate HIF-1a to upregulate PD-L1 remained unclear. Therefore, we reproduced the LPS-induced inflammatory environment in CRC to uncover the related mechanisms. We selected different concentrations of LPS (0, 0.1, 1, and 10 μM) to stimulate CT26 cells in order to screen the appropriate concentration of LPS for establishing the inflammatory environment. According to the results of WB, 10 μg/mL LPS was selected to build the inflammatory environment (Figure 3D,E). Further analyses indicated that the HIF-1α/PD-L1 pathway was activated in the LPS-induced inflammatory environment (Figure 3F,G).

LPS is a potent agonist of TLR4. Studies have shown that TLR4 activation is associated with PD-L1 expression [23]. To investigate TLR4 participation in HIF-1α-upregulated PD-L1 expression in CRC, TLR4, HIF-1α, and PD-L1 expression were further detected by WB in three paired CRC samples. We found that TLR4, together with HIF-1α and PD-L1, was negatively expressed in adjacent normal tissue but positively expressed in tumor tissue, implying a positive correlation between TLR4 and HIF-1α/PD-L1 (Figure 3H, I). These results were validated by tumor RNA-seq data from CRC samples in the TCGA dataset (Figure 3J). To explore whether the HIF-1α/PD-L1 expression induced by LPS in CT26 cells depends on TLR4, the TLR4 inhibitor C34 was used. As expected, C34 significantly suppressed increasing expression of HIF-1α/PD-L1 in the LPS-induced inflammatory environment (Figure 3K,L).

These results indicate that TLR4 can regulate HIF-1α/PD-L1 expression in the inflammatory environment of CRC.

### 2.4. Curcumin Suppressed Tumor Immune Escape through Inhibiting the TLR4/HIF-1α/PD-L1 Pathway

**Figure 4:**
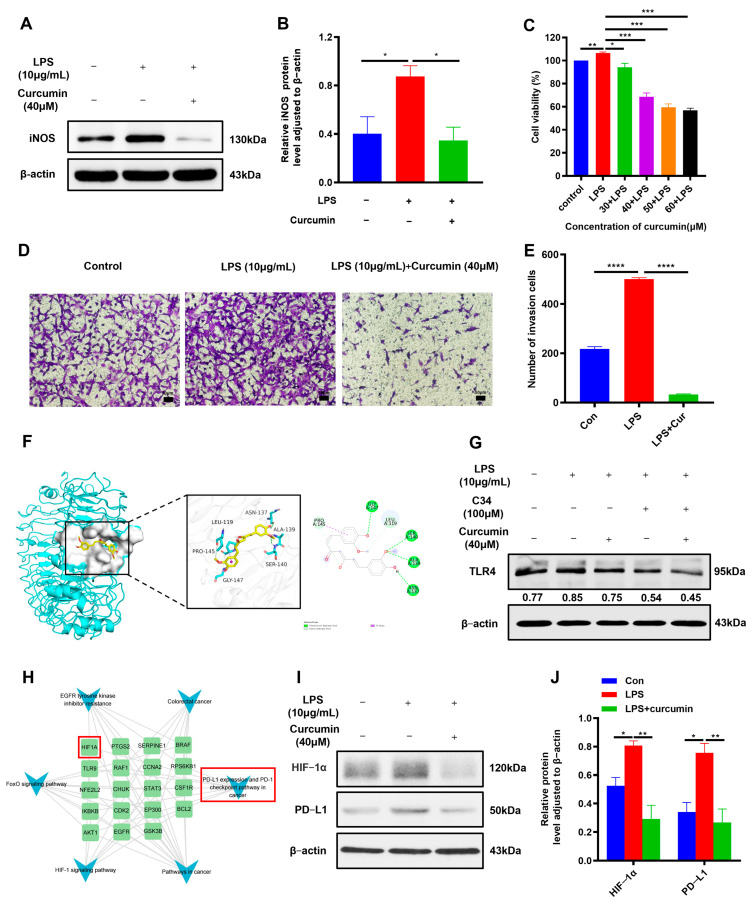
Curcumin inhibited immune escape by suppressing the TLR4/HIF-1α/PD-L1 pathway in CT26 cells. (**A**,**B**) The level of iNOS in CT26 cells in the presence or absence of LPS after curcumin (40 μM) treatment for 24 h (*n* = 3). (**C**) Cytotoxicity effect of curcumin on LPS-induced cell viability in CT26 cells (*n* = 6). (**D**,**E**) Migration effect of curcumin on LPS-induced cell viability in CT26 cells (scale bars = 50 μm) (*n* = 3). (**F**) Three-dimensional ribbon model of the curcumin–TLR4 complex. (**G**) The expression of TLR4 in CT26 cells exposed or not to LPS (10 μg/mL) after TLR4 inhibitor C34 (30 μM) or curcumin (40 μM) treatment for 24 h. One experiment out of three independent experiments is shown. (**H**) Network pharmacology revealed that curcumin mediated the “PD-L1 expression and PD-1 checkpoint pathway in cancer” pathway in the treatment of CRC. (**I**,**J**) The expression of HIF-1α/PD-L1 in CT26 cells in the presence or absence of LPS after curcumin (40 μM) treatment for 24 h (*n* = 3). * *p* < 0.05, ** *p* < 0.01, *** *p* < 0.001, **** *p* < 0.0001.

Curcumin is reportedly a potent anti-inflammatory molecule [24]. Thus, we examined whether curcumin can regulate the LPS-induced inflammatory environment of CRC. The inflammatory environment was established by stimulating CT26 cells with 10 μg/mL LPS, and then the cells were treated with 40 μM curcumin, with iNOS as an inflammatory indicator for investigation. The WB results showed that curcumin downregulated iNOS expression in the inflammatory environment of CRC (Figure 4A,B). In addition, to further evaluate the effects of curcumin on cell proliferation and migration in the inflammatory environment of CRC, MTT and Transwell assays were used. The results showed that curcumin inhibited cell proliferation and migration in the inflammatory environment established by LPS-stimulated CT26 cells (Figure 4C–E). It has been reported that curcumin can inhibit the TLR4 signaling pathway to exert anti-inflammatory effects [25,26]. To verify whether curcumin can inhibit TLR4 receptors, we first conducted theoretical calculations through molecular docking. The molecular docking assay showed that almost all parts of curcumin bound to the binding pocket of TLR4 consisting of ASN137, ALA139, SER140, GLY147, PRO145, and LEU119 (Figure 4F). Then, the results of WB also confirmed that curcumin can inhibit TLR4 expression in the inflammatory environment established by LPS-stimulated CT26 cells (Figure 4G). We next performed “targets–pathways” network analysis through network pharmacology to investigate the downstream signaling molecules of TLR4 responsible for mediating the inhibitory effects of curcumin. The network suggested that curcumin may affect the HIF-1α-mediated “PD-L1 expression and PD-1 checkpoint pathway in cancer” (Figure 4H). Further, WB results exhibited that curcumin was able to inhibit HIF-1α and PD-L1 expression in the inflammatory environment established by LPS-stimulated CT26 cells (Figure 4I,J), suggesting that curcumin suppressed tumor immune escape by inhibiting the TLR4/HIF-1α/PD-L1 pathway.

## 3. Discussion

We demonstrated that the microenvironment of CRC is associated with the expression of the HIF-1α/PD-L1 pathway. Curcumin inhibited the associated pathway. Although many studies have shown that HIF-1α and PD-L1 are involved in the rapid progression of CRC, whether HIF-1α and PD-L1 interact with each other in the microenvironment of CRC needs more studies. In the present study, we revealed a positive relationship between HIF-1α and PD-L1 in CRC. Meanwhile, the hypoxic and inflammatory environment of CRC activated the HIF-1α/PD-L1 pathway, thereby promoting the immune escape of CRC. Furthermore, curcumin inhibited the associated pathway and prevented the development of CRC.

Hypoxia is frequently observed in various solid cancers, including CRC. Studies have indicated that hypoxia markedly enhances PD-L1 expression in myeloid-derived suppressor cells, macrophages, dendritic cells, as well as tumor cells [27,28,29]. This process depends on HIF-1α signal transduction. For the first time, we demonstrated that HIF-1α upregulates PD-L1 expression in the hypoxic environment of CRC. Furthermore, it was demonstrated that the HIF-1α inhibitor YC-1 can downregulate the HIF-1α/PD-L1 pathway. This suggests a combinational therapeutic approach in which combining HIF-1α inhibitors with PD-L1 blockade could abrogate tumor hypoxia and enhance the immune system in individuals with cancer. Previous studies revealed that targeting the HIF-1α/PD-L1 pathway with PD-L1 antibodies combined with the HIF-1α inhibitor PX-478 improved the efficacy of anti-PD-1/PD-L1 for glioma [10]. But the efficacy of combination therapy in the treatment of CRC still needs to be further explored.

In addition to the hypoxic environment, the inflammatory environment is also an important feature of CRC. Previous studies have shown that the development of CRC is related to the inflammatory environment of the intestine [16]. LPS, as a classical pro-inflammatory mediator, changes intestinal barrier integrity through deregulating the expression of TJ proteins [30], ultimately causing damage to the gut barrier [31,32]. LPS has the potential to penetrate the circulatory system in pathological conditions, resulting in a significant elevation of the circulating LPS levels in patients with CRC [33]. The concentration of LPS is significantly elevated in CRC tissues compared to normal colorectal tissues [34]. Therefore, the inflammatory environment of CRC was mimicked by LPS in this study. Our data suggested that the LPS-induced inflammatory environment promoted the release of the inflammatory indicator iNOS. The LPS-induced inflammatory environment activates HIF-1α, thereby promoting the development of CRC [35]. Interestingly, we found that HIF-1α activation in the inflammatory environment of CRC similarly upregulated PD-L1. This result suggests that in addition to the hypoxic environment, the inflammatory environment may also lead to the activation of the HIF-1α/PD-L1 pathway in CRC.

The inflammatory signaling pathway associated with LPS-activated TLR4/NF-κB is pivotal in promoting cancer invasion and metastasis in humans, contributing to the poor survival rate observed in CRC [36,37]. LPS serves as the primary activator of TLR4 across various cancer types, including NSCLC and CRC. Our data showed that the expression level of TLR4 was markedly elevated in CRC tissues compared to adjacent normal tissues. Some studies shown that TLR4 activation in tumor cells can upregulate the expression of PD-L1 and contribute to tumor immune escape [38,39,40]. Using bioinformatics and WB, our study confirmed a positive association between TLR4 expression and HIF-1α/PD-L1 expression. Furthermore, our study also elucidated the mechanism by which TLR4 regulates the HIF-1α/PD-L1 pathway. This explains the hypothesis that the HIF-1α/PD-L1 pathway is activated in the inflammatory environment of CRC.

Accumulating evidence indicates that small-molecule monomers show potential in combating tumors [41]. For instance, sulforaphane demonstrates anti-tumor properties by regulating several signaling pathways [42], such as Nrf2/Keap1 [43], AKT1/HK2 [44], and NF-κB/MMP-9 [45]. Curcumin, a widely recognized anti-inflammatory small molecule, significantly contributes to tumor suppression by regulating multiple signaling pathways [46]. Curcumin has been reported to prevent liver cancer progression by inhibiting TLR4 signaling [47]. Similarly, curcumin can also reduce PD-L1 expression by inhibiting Stat3 phosphorylation, thus bolstering the immune response against tumors [48,49]. We found that curcumin suppressed tumor progression via the TLR4/HIF-1α/PD-L1 pathway, suggesting that curcumin may be a potent immunomodulator for treating CRC.

Our study also had some limitations. Firstly, only the mouse CRC cell line CT26 was used in the present study. Although valuable information has been obtained from the murine cell line, it is essential to recognize significant differences between mice and humans in terms of physiological structure, biological characteristics, and genetic disparities. Therefore, it is necessary to cautiously extrapolate these results to humans. Secondly, in addition to the inflammatory mediator LPS, cytokines and chemokines can also act as TME modulators [50,51]. Hence, further research is needed to fully elucidate the impact of the inflammatory environment constructed by other inflammatory mediators on the HIF-1α/PD-L1 axis and its effect on immune escape. Thirdly, combination therapies based on the HIF-1α/PD-L1 pathway need to be evaluated in animal models and clinical cases.

## 4. Materials and Methods

### 4.1. Reagents and Antibodies

Curcumin (cat. no. B20614) was purchased from Yuanye (Shanghai, China). CoCl_2_ (cat. no. C8661) was purchased from Sigma-Aldrich (St. Louis, MO, USA). DFO (cat. no. HY-B0988) and TLR4-IN-C34 (cat. no. HY-107575) were purchased from MedChemexpress (Monmouth Junction, NJ, USA). YC-1 (cat. no. Y274733) was purchased from Aladdin (Shanghai, China). LPS (cat. no. L8880), MTT (cat. no. M8180), and BCA protein detection kits (cat. no. PC0020) were purchased from Solarbio (Beijing, China). A nitric oxide assay kit (cat. no. S0021S) was purchased from Beyotime Biotechnology (Shanghai, China). ECL chemiluminescent reagent (cat. no. P10300) was purchased from NCM Biotech (Suzhou, China). Antibodies against HIF-1α (Rabbit mAb, cat. no. 36169) were purchased from Cell Signaling Technology (Danvers, MA, USA). Antibodies against PD-L1 (Rabbit pAb, cat. no. A1645), iNOS (Rabbit mAb, cat. no. A3774), HRP Goat Anti-Rabbit IgG (H + L) (cat. no. AS014), and HRP Goat Anti-Mouse IgG(H + L) (cat. no. AS003) were purchased from ABclonal (Wuhan, China). Antibodies against β-actin (Mouse mAb, cat. no. 2D4H5) were purchased from Proteintech (Wuhan, China). Antibodies against TLR4 (Rabbit pAb, cat. no. WL00196) were purchased from Wanleibio (Shenyang, China).

### 4.2. Bioinformatics Analysis

The RNA sequencing data of normal and tumor tissues of CRC were retrieved from the GTEx database (https://gtexportal.org/, accessed on 30 October 2022) and the TCGA database (https://portal.gdc.cancer.gov/, accessed on 30 October 2022) and were used to compare the expression of HIF-1α and PD-L1 between normal and tumor tissues of CRC. The RNA sequencing data of GSE44076 were obtained from the GEO database (http://www.ncbi.nlm.nih.gov/geo/, accessed on 10 November 2022) and were used to validate the expression of HIF-1α and PD-L1 between normal and CRC tumor tissues. In addition, the correlation between HIF-1α and PD-L1 and the correlation between TLR4 and HIF-1α/PD-L1 were measured using the tumor tissue sequencing data for CRC from the TCGA database. Data are summarized in Supplementary Tables S1–S3 and S10.

### 4.3. Patient Samples

The study adhered to the principles outlined in the Declaration of Helsinki, and informed written consent was obtained from all participants. The protocol of this study (approval number: 20210831YG) was approved by the Ethics Committee of China Medical University, and CRC tissue specimens were collected from the Cancer Hospital of China Medical University. None of the patients received any medications before sample collection. All samples were kept at −80 °C. The detailed information on patients is listed in Table S4.

### 4.4. Cell Line and Cell Culture

The CRC cell line (CT26) was purchased from iCell Bioscience Inc (Shanghai, China). CT26 cells were cultured in normal culture medium, namely, DMEM supplemented with 10% FBS and antibiotics and 1% penicillin/streptomycin, in a humidified atmosphere of 5% CO_2_ at 37 °C.

### 4.5. Western Blotting

Protein samples were prepared from patient samples or cultured cells. Briefly, patient samples were homogenized in the homogenization buffer (EDTA 2 mM and HEPES 20 mM, pH = 7.5, containing aprotinin 1 μg/mL and PMSF 1 mM), then centrifuged at 10,000 rpm and 4 °C for 10 min. Cultured cells were gently washed with PBS and lysed for 30 min on ice using RIPA lysis buffer containing 1 mM PMSF and 1 μg/mL aprotinin. Then, the lysates were transferred to Eppendorf tubes and centrifuged at 10,000 rpm and 4 °C for 10 min. The supernatants were taken and kept at −20 °C for further experiments.

The same amounts of proteins were subjected to electrophoresis using SDS-PAGE after SDS sampling. Then, the proteins were transferred onto PVDF membranes from gel. Subsequently, the membranes were blocked with 5% nonfat milk at room temperature for 1 h, followed by incubation with appropriate primary antibodies against HIF-1α (1:1000), PD-L1 (1:1000), iNOS (1:1000), TLR4 (1:2000), or β-actin (1:1000) at 4 °C overnight. Membranes were then rinsed with TBST and incubated with HRP-conjugated secondary antibodies (1:5000) at room temperature for 1 h. Finally, the blotted membranes were visualized with ECL chemiluminescent reagent and detected with X-ray film and a gel imaging system. All WB experiments were repeated three or more times.

### 4.6. Immunohistochemistry

IHC staining was performed on the patients’ samples. Briefly, sections were deparaffinized in xylene and subjected to two washes, then rehydrated with successive washes in 100%, 95%, 85%, and 75% graded ethanol. After antigen retrieval was performed with citrate buffer (pH 6) in a pressure cooker, these sections were placed in a pressure cooker and fixed for 2 min. A quantity of 3% hydrogen peroxide was used to block endogenous peroxidase activity for 20 min. The sections were blocked with 10% goat serum for 30 min at 37 °C. Afterwards, the samples were incubated with the primary antibody, anti-HIF-1α (1:200, cat. no. bs-0737R, Bioss, Beijing, China), anti-PD-L1 (1:300, cat. no. 66248-1-Ig, Proteintech), or anti-TLR4 (1:600, cat. no. GB11519-100, Servicebio, Wuhan, China), at 4 °C overnight. Next, the sections were washed, then incubated with the secondary antibody for 30 min at room temperature, the secondary antibodies being conjugated to HRP. DAB was used as a chromogen, and sections were stained with hematoxylin, and after a short while a microscope was used to determine the staining intensities.

### 4.7. MTT Assay

The cytotoxicity of curcumin and LPS in CT26 cells was assessed using the MTT assay. CT26 cells were seeded at a density of 5 × 10^3^ cells/well in 96-well plates. Cells were exposed to varying concentration of curcumin and 10 μg/mL LPS for 24 h. Following treatment, 20 µL of MTT solution (5 mg/mL) was introduced into each well and incubated for an additional 4 h. Then, the medium was aspirated, and DMSO (150 µL) was added, followed by gentle shaking for 10 min to dissolve the formazan crystals. The absorbance of the solution was measured using a microplate reader at 490 nm. Cell viability was calculated using the following formula: ratio (%) = [OD (Curcumin) − OD (Blank)]/[OD (Control) − OD (Blank)] × 100%. Data are summarized in Supplementary Table S9.

### 4.8. Transwell Assays

For the cell migration experiment, 5 × 10^4^ CT26 cells in the serum-free medium (100 μL) were added to the upper chamber in the presence of LPS with or without curcumin. Meanwhile, 600 μL of normal culture medium was added to the lower chamber. After incubation at 37 °C for 24 h, cells remaining in the upper chamber of the membrane were removed using a cotton swab, fixed with 4% paraformaldehyde, and stained using 0.1% crystal violet. Cells migrating from the upper to the lower chamber of the filter were counted using a light microscope.

### 4.9. Molecular Docking

Autodock vina was used for molecular docking, and the binding mode between curcumin and the TLR4 protein was studied. The structure of TLR4 was stored in the protein data bank (PDB) (https://www.rcsb.org/, accessed on 15 August 2023), and the TLR4 PBD ID is 3FXI. Curcumin was treated with ligand preparation and minimizing model processing in AutodockTools 1.5.6.

### 4.10. Network Pharmacology

#### 4.10.1. Identification of Potential Targets of Curcumin

Curcumin targets were obtained by searching SwissTargetPrediction (http://www.swisstargetprediction.ch/, accessed on 29 October 2023) and DrugBank (https://www.drugbank.com/, accessed on 29 October 2023). Targets were identified by searching the PubMed database. The identified targets were cross-referenced with gene names using the UniProt database (https://www.uniprot.org/, accessed on 29 October 2023). Subsequently, the obtained target genes were amalgamated, and any duplicates were removed. The results were considered as the potential targets of curcumin. Data are summarized in Supplementary Table S5.

#### 4.10.2. Prediction of Potential Targets of CRC

CRC targets were searched and obtained from GeneCards (https://www.genecards.org/, accessed on 30 October 2023), the Therapeutic Target Database (TTD) (https://db.idrblab.net/ttd/, accessed on 30 October 2023), and Online Mendelian Inheritance in Man (OMIM) (https://www.omim.org/, accessed on 30 October 2023). The identified targets were cross-referenced with gene names using the UniProt database (https://www.uniprot.org/, accessed on 30 October 2023). Subsequently, the obtained target genes were amalgamated, and any duplicates were removed. The results were considered the potential targets of CRC. Data are summarized in Supplementary Table S6.

#### 4.10.3. Curcumin–CRC Common Target Screening

Curcumin targets and CRC targets were inputted into VENNY 2.1 (https://bioinfogp.cnb.csic.es/tools/venny/, accessed on 30 October 2023) to acquire common targets. Data are summarized in Supplementary Table S7.

#### 4.10.4. Construction of a Target–Pathway Network

Curcumin–CRC intersection targets were imported into DAVID (https://david.ncifcrf.gov/, accessed on 30 October 2023) to perform enrichment analysis of the KEGG pathway. Cytoscape3.8.2 (https://cytoscape.org/, accessed on 29 April 2023) was used to construct a network and visualize the top-ranked possible pathways. Data are summarized in Supplementary Table S8.

### 4.11. Statistical Analyses

Statistical analyses were conducted utilizing GraphPad Prism 8.0. Data are presented as means ± SEs. A non-paired Student’s *t*-test or ANOVA was used to compare the differences between two or more groups. *p* < 0.05 was considered statistically significant.

## 5. Conclusions

**Figure 5:**
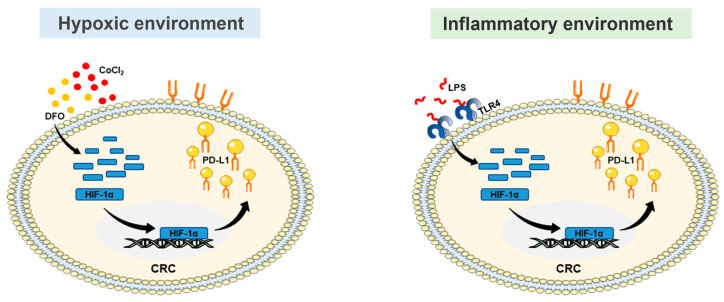
Hypoxic and inflammatory environment activated the HIF-1α/PD-L1 pathway.

Our findings strongly suggest that the hypoxic and inflammatory environment mediates the HIF-1α/PD-L1 pathway to promote immune escape in CRC (Figure 5). Furthermore, curcumin could inhibit immune escape by suppressing the TLR4/HIF-1α/PD-L1 pathway in the inflammatory environment of CRC.

## Data Availability

Data will be made available on request.

## Supplementary Materials

The supporting information can be downloaded at: https://www.mdpi.com/article/10.3390/ijms25073735/s1.
